# Lifting the burden of headache in China: managing migraine in a SMART way

**DOI:** 10.1186/s10194-017-0790-6

**Published:** 2017-08-08

**Authors:** Shengyuan Yu, Timothy J Steiner

**Affiliations:** 1 Department of Neurology, Chinese PLA General Hospital, Fuxing Road 28, Haidian District, Beijing, 100853 China; 2 Department of Neuroscience, Norwegian University of Science and Technology (NTNU), Trondheim, Norway; 3 Division of Brain Sciences, Imperial College London, London, UK

**Keywords:** China, Migraine, Headache schools, Smart, Continuing medical education, *Lifting the burden*, Global campaign against headache

## Abstract

With support from *Lifting The Burden* , a UK-registered charitable organization, a nationwide survey of headache disorders in the Chinese adult population was conducted in 2008–2009. This project, which was within the Global Campaign against Headache, showed that headache disorders have a major adverse impact on public health in China. Subsequently, as essential support for implementing headache services around the country, an enactment of stage 3 (intervention) of the Global Campaign against Headache − the continuing medical education (CME) program *Headache Schools −* was established. ‘SMART’ (Screen, Migraine, Aura, Red flag and Treatment), a systematic and operational disease management model, was introduced with the aims of enhancing neurologists’ knowledge of migraine, standardizing their diagnostic and treatment approaches, and improving their practices and outcomes. To date, 615 neurologists have been trained and 135 headache clinics have been established. In future, as we promote SMART in CME, we can use the database of our computerized clinical decision support systems to evaluate the impact on treatment outcomes.

## Correspondence/findings

In this letter, we present the current situation of headache disorders in China and the continuing medical education (CME) programs carried out to improve disease treatment. Specially, the systematic disease management model “SMART” was introduced to standardize migraine management. Numbers of neurologists were benefited from these series of CME and SMART model were well accepted and implemented in their clinical practice.

The Global Campaign against Headache was launched in 2003 by *Lifting The Burden* , a UK-registered nongovernmental organization working in collaboration with the World Health Organization. Its clear ultimate purpose, to reduce the burden of headache worldwide [1], was formulated in stages: to improve knowledge of the burden of headache throughout the world, raise public and political awareness of it, and support the implementation of activities to relieve it in countries everywhere. The Campaign has achieved notable results: headache disorders are now recognized as the third leading cause of disability globally [2, 3].

With support from *Lifting The Burden* , we conducted a population-based, door-to-door survey of adults in the Chinese population during 2008–2009, which estimated the 1-year prevalence of migraine at 9.3% [4]. Despite this evidence – that nearly 1 in 10 Chinese adults experienced migraine every year – the survey found that under-diagnosis and misdiagnosis were common. Only half (52.9%) of people with migraine had consulted a doctor specifically for headache. More tellingly, fewer than 1 in 7 (13.8%) were correctly diagnosed [5, 6]. Only 2.7% of people with migraine had been given preventative medication [6]; in the United States, the expected prophylaxis proportion, based on a large epidemiological investigation [7], was around 25%.

A finding of the survey was that there is a large gap between the medical needs of the Chinese population with migraine and the diagnostic and treatment skills of, among others, neurologists who manage headache in China [8]. In 2015, a continuing medical education (CME) program ( *Headache Schools* ) was initiated at the Chinese PLA General Hospital in Beijing. More than 200 neurologists who expressed a speciality interest in headache management attended the first program, which was supported by the International Headache Society. As part of the educational activities and clinical practice training, a systematic and operational disease management model known as ‘SMART’ (Screen, Migraine, Aura, Red flag and Treatment) was introduced to standardize clinical diagnosis and treatment approaches to migraine. This disease management model has provided opportunities for practitioners to enhance their knowledge of primary headaches, especially migraine, and use this knowledge to improve their daily practice and clinical outcomes.

SMART integrates the screening, diagnosis, and treatment of migraine. The word “SMART” serves as a reminder of the components of migraine management. The Screen element of the model emphasizes the use of validated scales to recognize possible migraine among patients with headache – the first step in successful management. The ID-Migraine screening instrument for migraine, which has been reported to have a sensitivity of 87.5% and specificity of 100% in a general population [9], has now been validated in a Chinese population [10].

The Migraine element of SMART emphasizes that, whenever migraine is a possibility, diagnostic confirmation according to the criteria of ICHD-3-beta [11] is needed, and this requires careful assessment. The Aura element guides neurologists through key diagnostic points in their conversations with patients, particularly to identify migraine with aura.

Neurologists must always consider differential diagnoses, and remain alert to other possible diagnoses that raise a need for further examinations: the Red flag element of SMART serves mostly to signal cases that might be secondary headache. Once the diagnosis of migraine is confirmed, neurologists should consider, then offer, the most appropriate treatments. For patients with migraine, with or without aura, these extend beyond non-pharmacological treatments and acute pharmacological therapies for acute attacks.

**Fig. 1:**
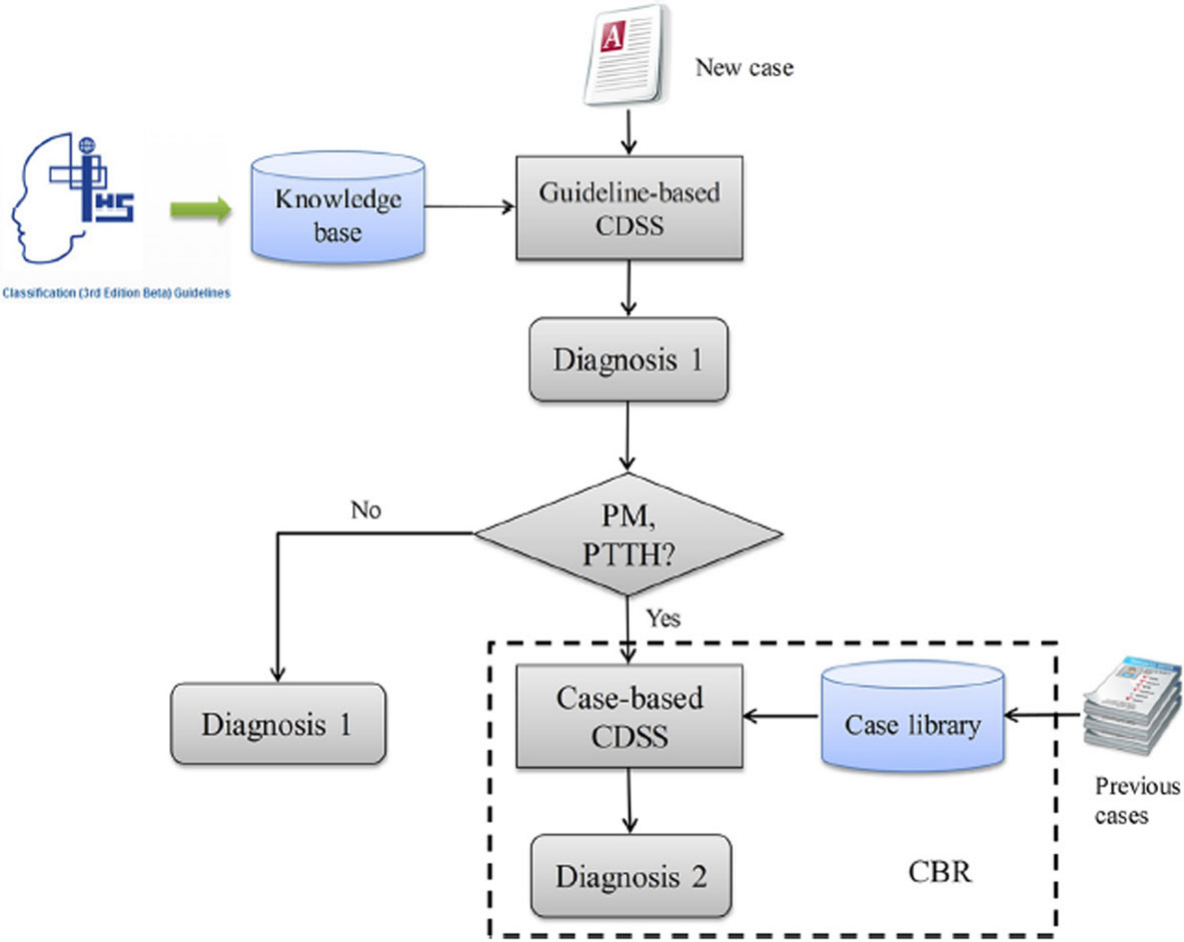
A computerized clinical decision support system (CDSS) [from reference 13 ]. CBR, case-based reasoning; PM, probable migraine; PTTH, probable tension-type headache

The treatment element of SMART emphasizes prophylaxis, since this is an important but neglected part of management for many patients, and is helpful in preventing chronicity and acute therapy overuse. However, more education is needed, because many general practitioners and even neurologists at primary care and secondary care levels find the relative complexity of some headache subtypes and their diagnostic criteria confusing. As reported earlier [12, 13], we have also developed a computerized clinical decision support system (CDSS) [Fig. 1], which is based on ICHD-3-beta [11], and has sensitivity and specificity for migraine without aura of >99% and >97%, respectively, and for migraine with aura of 100% and 100%, respectively [12, 13]. In headache clinics, healthcare providers have adopted CDSSs in combination with SMART to optimize their management of headache disorders, especially migraine.

**Fig. 2:**
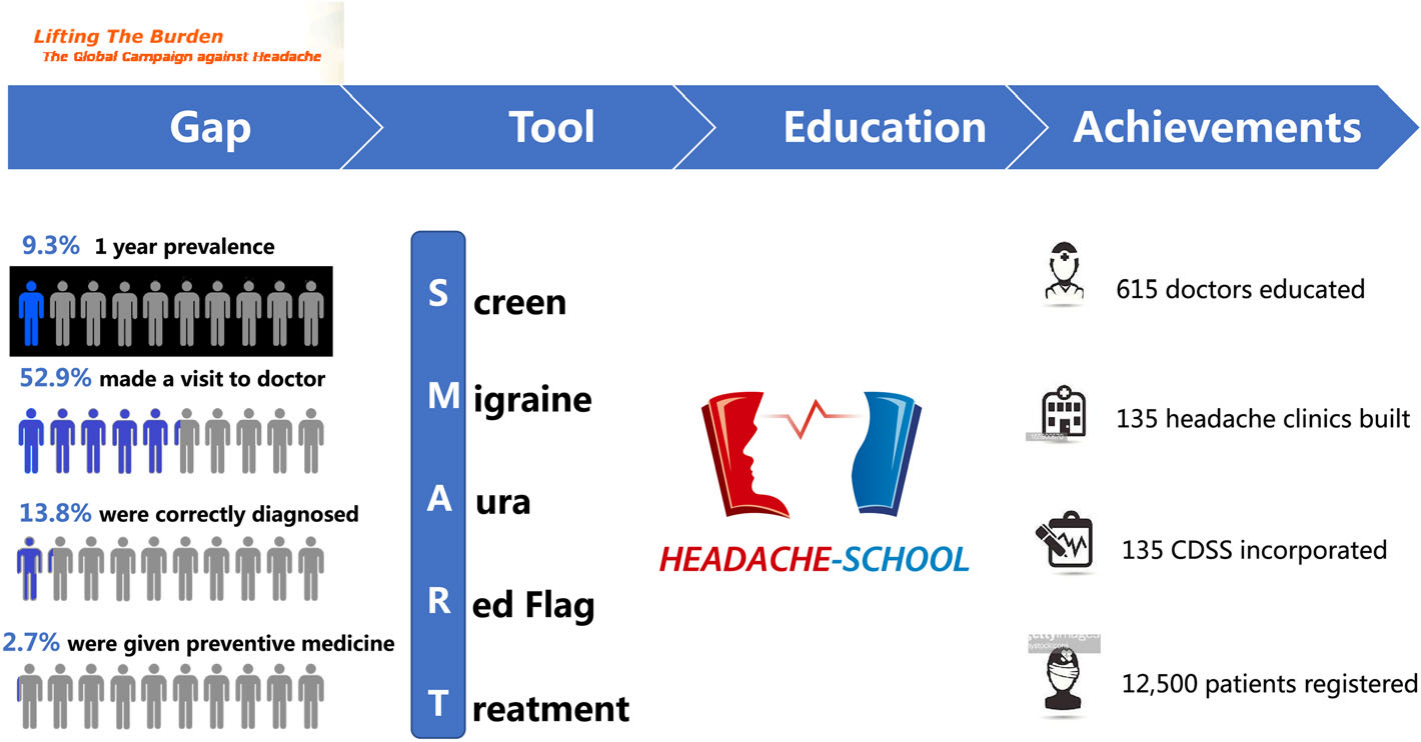
The process and achievements of SMART

So far, the Headache Schools CME program has been held 6 times, training a total of 615 doctors from urban and rural communities. For these doctors who have been trained, they will offer training in this field to other health-care providers. Nationwide, 135 headache clinics have been established and have implemented CDSSs, in which the medical records of around 12,500 patients have now been included. The Headache Schools program and SMART model have been shared at several national academic congresses, and are now well accepted by Chinese neurologists. These achievements are illustrated in Fig. 2 . The train-the-trainer model is confirmed as a virtuous management approach in headache disorders, also in line with other activities endorsed by Lifting The Burden, such as Master in Headache Medicine at Sapienza University of Rome. Till 2015, 119 physicians from all over the world have accepted the one-year course of training in excellence and acquainted their Master Degree in Headache Medicine. The master degree physicians were offering education and raising awareness locally of the burden and treatment of headache [14, 15].

In future years, as we continue this CME program and implement the SMART model in clinicians’ practice, we can analyze the clinical data in CDSSs to evaluate the impact on clinical practice and treatment outcomes. We hope that this enactment of stage 3 (intervention) of the Global Campaign against Headache [1] will demonstrate educational and practical benefits to neurologists and, more importantly, measurable health benefits for their patients with headaches.
